# Factors determining the knowledge and prevention practice of healthcare workers towards COVID-19 in Amhara region, Ethiopia: a cross-sectional survey

**DOI:** 10.1186/s41182-020-00254-3

**Published:** 2020-08-20

**Authors:** Mulusew Andualem Asemahagn

**Affiliations:** School of Public Health, College of Medicine and Health Sciences, Bahir Dar University, Bahir Dar, Ethiopia

**Keywords:** COVID-19, Knowledge, Practice, Healthcare workers, Amhara region, Ethiopia

## Abstract

**Background:**

Healthcare workers (HWs) are at the highest risk of getting CIVID-19. This study aimed to assess factors determining the knowledge and prevention of HWs towards COVID-19 in the Amhara Region, Ethiopia.

**Methods:**

A cross-sectional online survey was conducted among 442 HWs using email and telegram addresses. The knowledge and practice of HWs were estimated using 16 knowledge and 11 practice questions. A multivariable logistic regression analysis was used on SPSS version 25 to identify factors related to the knowledge and prevention practice of HWs on COVID-19. Significance was determined at a *p* value of < 0.05 and association was described by using odds ratio at 95% CI.

**Results:**

Of 442 HWs, 398 (90% response rate) responded to the online interview questionnaire. From 398 HWs, 231(58%), 225(56%), 207(53%), and 191(48%) were males, from rural area, aged ≥ 34 years and nurses, respectively. About 279(70%) HWs had good knowledge of COVID-19 followed by 247(62%) good prevention practices. Age < 34 years (AOR = 2.14, 95% CI = 1.25–3.62), rural residence (AOR = 0.44, 95% CI = 0.26–0.70), access to infection prevention (IP) training (AOR = 2.4, 95% CI = 1.36–4.21), presence of IP guideline (AOR = 2.82, 95% CI = 1.64–4.62), and using social media (AOR = 2.51, 95% CI = 1.42–4.53) were factors of knowledge about COVID-19. Whereas, rural residence (AOR = 0.45, 95% CI = 0.31–0.75), facility type (AOR = 0.40, 95% CI = 0.28–0.89), access to IP training (AOR = 2.32, 95% CI = 1.35–4.16), presence of IP guidelines (AOR = 2.10, 95% CI = 1.21–3.45), knowledge about COVID-19 (AOR = 2.98, 95% CI = 2.15–5.27), having chronic illnesses (AOR = 2.0, 95% CI = 1.15–3.75), lack of protective equipment (PPE) (AOR = 0.42, 95% CI = 0.32–0.74), and high workload (AOR = 0.40, 95% CI = 0.36–0.87) were factors of COVID-19 prevention.

**Conclusion:**

In this study, most of the HWs had good knowledge but had lower prevention practice of COVID-19. Socio-demographic and access to information sources were factors of knowledge on COVID-19. Similarly, residence, shortage of PPE, high workload, comorbidities, knowledge, and access to IP training and guideline were factors limiting prevention practices. Thus, a consistent supply of PPE and improving health workers’ knowledge, making IP guidelines and information sources available, and managing chronic illnesses are crucial to prevent COVID-19 among HWs.

## Background

World Health Organization (WHO) declared that the coronavirus 2019 (COVID–19) as a pandemic on 11 March 2020, after 11 days of being declared as a public health emergency [1, 2]. The COVID-19 has been reported as a continuing global epidemic since its first appearance in December 2019 from Wuhan City in China [2, 3]. The COVID-19 is a zoonotic contagious disease that can transmit from animal to human and from human to human [4]. The major transmission route of COVID-19 is respiratory droplets produced from an infected person while sneezing and coughing. It is also transmitted by infected surfaces and objects since the virus can survive everywhere [3, 5, 6]. The COVID-19 has been characterized by wide clinical futures ranging from no symptoms to a severe form of respiratory illness [7–9]. The typical signs and symptoms of COVID-19 include respiratory symptoms, fever, cough and shortness of breath [4, 6–8, 10]. Occasionally, symptoms including headache, muscle pain, sore throat, loss of taste or smell, hemoptysis, and diarrhea were observed [9, 11].

The burden of COVID-19 has increased worldwide in terms of morbidity, mortality and economic crisis [2, 12, 13]. Globally, as of 27 July 2020, over 16 249 165 confirmed cases of COVID-19 and 649 208 deaths were reported [14]. Although the spread of COVID-19 is highest in Europe and America, it has been alarmingly increased in Africa [13, 15–17]. The situation might be serious in Sub-Saharan Africa due to high comorbidities (HIV, TB and malaria), poverty, and poor healthcare service quality and access to health facilities [13, 15]. As of 27 July 2020, 847,628 confirmed cases and 17,759 deaths were reported from Africa. The situation has no exception in Ethiopia, where the burden of COVID-19 has increased and about 13,968 confirmed cases and 223 deaths have been reported as of 27 July 2020 [14].

HWs are the highest risk groups for COVID-19 due to the nature of their occupation that exposed them to infectious people with COVID-19 every day. Several HWs have infected by COVID-19 and lost their lives globally due to job-related COVID-19 [12, 18, 19]. Unless special attention is given to make HWs and their working places safe, the system will lose many HWs and highly compromise the capacity of anti-COVID-19 and other infectious diseases worldwide. Unlike other people, the HWs have double sources of infection to COVID-19 from the community and working places. The main reasons for acquiring COVID-19 among HWs include long-time exposure, shortage and poor quality of PPE [18, 20]. The HWs are typical infection sources of families, patients and the community [15, 20, 21].

To date, much is known about the distribution, transmission, prevention, and supports, but no curative treatment or vaccine that has been recommended for the COVID-19 [1, 6, 8, 22]. The WHO recommends the prevention of human-to-human transmission by avoiding close contacts, frequent handwashing with soap, and/or alcohol-based hand rubbing sanitizer, wearing PPE (facemask, shields and glove) and avoid going to crowded places [10, 22, 23]. Also, improving the knowledge and prevention practice of HWs and the community through regular updates about COVID-19 is crucial [10, 23]. If HWs have access to information sources, they will upgrade their knowledge and apply preventive devices to prevent COVID-19 and give appropriate care to patients, families and the community [15, 18, 23].

Recent literature on infection prevention (IP) practice of HWs in Ethiopia also depicted the presence of relatively better knowledge and attitude on infection prevention practices. The prevention practice of most HWs however did not go with their knowledge and attitude levels [21, 24–26]. This might be related to less attention to IP and work safety, absence and poor quality of PPE, negligence of HWs and less comfortable working offices. Also, there is no recent evidence on the existing prevention practice of HWs towards COVID-19 in Ethiopia, in particular, the Amhara Region. Thus, this study aimed to assess the prevention practice and associated factors of HWs towards COVID-19 in the Amhara Region, Ethiopia. This might play a vital role in preventing COVID-19 among HWs and stop the spread of infections to the community.

## Methods

### Study design and settings

Due to the country’s lockdown for COVID-19 prevention, an online cross-sectional study was conducted between April and May 2020 among HWs working in public hospitals and health centers (HCs) of the Amhara Region, Ethiopia. Amhara Region is the second-largest region in Ethiopia. Amhara Region is divided into 10 administrative zones (third administration level in Ethiopia) and 3 town administrations. The capital city of the region is Bahir Dar city, where the regional health bureau and Amhara regional Public Health Institute are located. Based on the 2018 regional health bureau report, the region has about 4267 public health facilities (77 hospitals, 848 HCs, and 3342 health posts) to offer healthcare services to a total population of 21,841, 999:4,089,997 urban and 17,752,002 rural. A total of 38,000 HWs with different professional disciplines are working in those healthcare facilities [27].

### Sample size determination and sampling procedures

The sample size of the study participants (442) was determined using a single population proportion formula based on the following assumption: 50% proportion to prevention practice among HWs since no previous study on COVID-19 prevention practice, 95% confidence level, 5% margin of error, and 15% non-response rate by considering high delayed responses and non-respondents since it is an online survey. The study participants were selected randomly from the alphabetical list of all HWs in the Amhara Regional Health Bureau using the Stat Trek Random Number Generator tool [28]. Then, the investigator addressed sampled HWs through the regional health bureau, zonal health departments and human resource managers of health facilities. Based on the selected HWs, 70 health facilities (10 hospitals and 60 health centers) were study sites.

### Data collection tools and techniques

Data were collected online using a structured questionnaire and using email and telegram services of HWs working in different units of hospitals and HCs. The questionnaire was designed using Google Forms (via docs.google.com/forms) by referring to former studies on IP and the WHO IP guidelines [15, 16, 18, 23, 26, 29]. The questionnaire consists of questions related to demographics, information sources, risk assessment, knowledge and practice towards the COVID-19. The clarity, appropriateness and redundancy of questions were revised based on findings from the pretest. HWs had been informed well about the purpose of the study, data confidentiality and data collection procedures. After they became clear about the study and its procedure, the investigator asked each participant for consent by sending the consent form before data collection. After collecting the signed consent form from each health worker, the investigator sent the Google form link (questionnaire) to HWs for data collection. Data were collected from 5 April to 25 May 2020. In this study, HWs are health professionals who had primary contact with patients during clinical examination and biological specimen collection that include physicians, nurses, health officers and laboratory technicians/technologists.

### Data quality assurance

The questionnaire was designed with ease of use and pretested before data collection. Cronbach alpha was used to check the validity of the tool and the value of ‘α’ was 8.92. HWs had been informed of detailed information with practice on how to complete and sent the questionnaire. Duplication of responses was controlled by restricting to one response. The incompleteness of responses was reduced by making each “*required” to pass to the next question.

### Data management and analysis

The collected data were checked for completeness and exported to the MS-excel format. The excel data were then exported to SPSS version 25 for editing and analysis. There were 16 knowledge questions with “yes = 1” or “no = 0” responses to give values ranging from 0 to 16. A health worker who scored 80% and above was grouped as having “good knowledge” and who scored below 80% was grouped as having “poor knowledge.” On the other hand, there were 11 practice-related questions responded as “always = 1” and “rarely = 0” with total values ranging from 0 to 11. A health worker who scored 75% and above was grouped as “good practitioner” and who scored below 75% was grouped as “poor practitioner” [15]. The reason for using a 75% cut off value for practice was by considering the seriousness of the COVID-19, and the study participants are health workers to whom the prevention practice is mandatory to keep themselves families safe from COVID-19 and be a role model to their patients and the rest of the community.

Descriptive statistics including mean, median, standard deviation, range, cross-tabulations and proportions were computed. The model fitness was checked by the Hosmer-Lemeshow goodness of fit test before the regression analysis. Bivariate and multivariable logistic regression analyses were conducted to identify factors associated with HWs’ knowledge and practice towards COVID-19. Variables with a *p* value of < 0.2 in the bivariate analysis were used to fit the multivariable model to control the confounding effect. Variables with a *p* value of < 0.05 in the multivariable model were considered as significant factors. Associations between study and outcome variables were described using the odds ratio at 95% CI.

## Results

### Socio-demographic and risk assessment of health workers

Of the total 442 HWs, 398(90% response rate) responded to the online survey interview and 231(58%) were males. Over half, 207(53%) HWs, aged ≥ 34 years (mean age = 34 ± 5 years). Over half, 225(56%) HWs, were working in rural health facilities. Nearly half, 191(48%), of the HWs were nurses and 243 (61%) were from HCs. Only 88(22%) HWs had histories of domestic travel in recent times. A small number of HWs, 60 (15%), 48 (12%) and 179 (45%), had histories of chronic illness, smoking and taking alcohol in any amount, respectively. A limited number of HWs, 151 (38%) took training in IP in recent times. A significant number of HWs, 239 (60%) and 259 (65%), used social media and television and or radio as information sources about COVID-19, respectively. Over half, 207(52%), of the HWs noted the presence of adequate PPE in their health facilities. However, only 159 (40%) HWS stated the presence of IP guidelines in their working areas. Less than half, 167(42%), of the HWs reported as high workload prevented them from practicing COVID-19-prevention (Table 1).

**Table 1:** Socio-demographic characteristics of HWs in the Amhara Region, Ethiopia, 2020

Variable	Response	Frequency (%)
Age in years	< 34	187 (47.0)
	≥ 34	211 (53.0)
Sex	Male	231 (58.0)
	Female	167 (42.0)
Profession	Physician	32 (8.0)
	Nurse	191 (48.0)
	Health officer	60 (15.0)
	Midwifery	64 (16.0)
	Laboratory	51 (13.0)
Residence	Rural	225 (56.0)
	Urban	173 (44.0)
Marital status	Single	119 (30.0)
	Married	271 (68.0)
	Divorced	8 (2.0)
Family size	≤ 4	287 (72.0)
	> 4	111 (28.0)
Working experience in years	≤ 5	159 (40.0)
	> 5	239 (60.0)
HWs’ health facilities	Health centers (HCs)	243 (61.0)
	Hospital	155 (39.0)
Trained in IP within a year	Yes	151 (38.0)
	No	247 (62.0)
Have travel history in recent times	Yes	40 (10.0)
	No	358 (90.0)
Have chronic illnesses	Yes	60 (15.0)
	No	338 (85.0)
Smoke cigarette in any amount	Yes	48 (12.0)
	No	350 (82.0)
Drinking alcohol in any amount	Yes	179 (45.0)
	No	219 (55.0)
Use social media as an information source	Yes	239 (60.0)
	No	159 (40.0)
Television /radio is my information source	Yes	259 (65.0)
	No	139 (35.0)
Adequate access to PPE in health facilities	Yes	207 (52.0)
	No	191 (48.0)
Adequate access to disinfectants	Yes	112 (28.0)
	No	286 (72.0)
There is IP guideline in health facilities	Yes	159 (40.0)
	No	239 (60.0)
High workload lowered my IP practices	Yes	167 (42.0)
	No	231 (58.0)
Discomfort while using PPE lower my utilization	Yes	207 (52.0)
	No	191 (48.0)

### Knowledge of health workers about COVID-19 infection

**Fig. 1:**
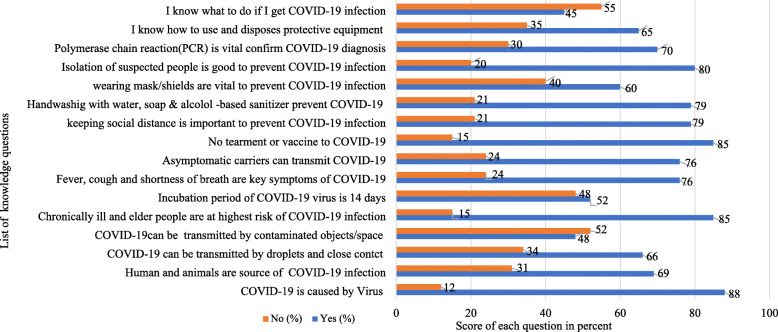
Knowledge of HWs about COVID-19 in the Amhara region of Ethiopia, 2020

Of the surveyed HWs, 279(70%) had demonstrated good knowledge about COVID-19. Most, 351(88%) and 339(85%) of the HWs reported that COVID-19 is a viral disease and has no effective treatment or vaccine yet, respectively. Over two-thirds, 275(69%) HWs stated as animals and humans are the primary sources of infection to COVID-19. Also, 263(66%) HWs mentioned respiratory droplets and close contact are the main transmission routes of COVID-19. Nearly half, 191(48%) HWs, also reported contaminated objects and surfaces as potential transmission routes. The majority, 338 (85%) HWs identified chronically ill people are at the highest risk of COVID-19. In addition, 303(76%) HWs pointed out that fever, dry cough and shortness of breath are typical signs and symptoms of people who had COVID-19. Also, 318(80%) and 315(79%) HWs knew that frequent handwashing and social distance are important to prevent COVID-19. Moreover, 85% and 80% HWs mentioned that COVID-19 had no cure treatment or vaccine, and isolation of suspected people is crucial to prevent COVID-19, respectively (Fig. 1).

### The COVID-19 prevention practices of health workers

**Fig. 2:**
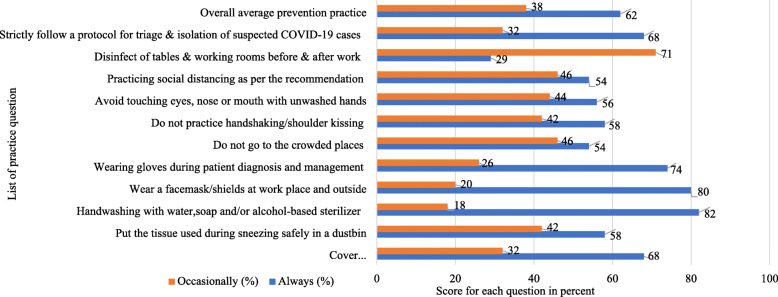
The prevention practice of HWs towads COVID-19 in the Amhara region of Ethiopia, 2020

In this study, 247(62%) HWs had good prevention practices towards COVID-19. The majority, 326(82%) and 318(80%) HWs regularly practice handwashing or alcohol-based sanitizer and wearing facemasks, respectively. Similarly, 271(68%) HWs frequently cover their mouth and nose while sneezing and 231(58%) of them disposed of the covering materials they used during sneezing properly to the dustbin. Also, 231(58%), 223(56%), and 215(54%) HWs avoid handshaking/shoulder kissing/touching mouth/nose/eye with unwashed hands and go to the crowded places, respectively. Only 116(29%) HWs always use disinfectants (Fig. 2).

### Factors associated with HWs’ knowledge about COVID-19

Based on the multivariable logistic regression model, HWs < 34 years of age were double times to have good knowledge about COVID-19 compared to people aged 34 years and above (AOR = 2.14, 95% CI = 1.25–3.62). HWs from rural health facilities were 56% times less likely to have good knowledge about COVID-19 compared to the counterpart HWs (AOR = 0.44, 95% CI = 0.26–0.70). Similarly, the odds of having good knowledge among HWs who got training in IP was over twice than the counterpart HWs (AOR = 2.4, 95% CI = 1.36–4.21). HWs who used social media as information sources were 2.51 times knowledgeable compared to HWs who did not access information using social media (AOR = 2.51, 95% CI = 1.42–4.53). The odds of having good knowledge among HWs who had access to IP guideline was nearly three times more compared to the counterpart HWs (AOR = 2.82, 95% CI = 1.64–4.62) (Table 2).

**Table 2:** Factors affecting HWs’ knowledge of COBID-19 in the Amhara Region, Ethiopia, 2020

Variables	Knowledge on COVID-19		COR (95% CI)	AOR (95% CI)
	Good	Poor		
Age				
< 34	150 (37.7)	37 (9.3)	2.58 (1.64–4.06)	2.14 (1.25–3.62)
≥ 34	129 (32.4)	82 (20.6)	1	1
Sex				
Male	156 (39.2)	75 (18.8)	0.74 (0.48–1.16)	0.60 (0.25–1.06)
Female	123 (31.0)	44 (11.0)	1	1
Residence				
Rural	140 (35.1)	85 (21.4)	0.41 (0.25–0.64)	0.44 (0.26–0.70)
Urban	139 (35.0)	34 (8.5)	1	1
Family size				
≤ 4	197 (49.5)	90 (22.6)	0.77 (0.47–1.27)	0.56 (0.24–1.15)
> 4	82 (20.6)	29 (7.3)	1	1
Working experience				
≤ 5 years	115 (29.0)	44 (11.0)	1.20 (0.77–1.86)	0.86 (0.46–1.24)
> 5 years	164 (41.2)	75 (18.8)	1	1
HWs’ health facility				
Health center	154 (38.7)	89 (22.3)	1.15 (0.76–1.75)	0.85 (0.52–1.45)
Hospital	93 (23.4)	62 (15.6)	1	1
Trained in infection prevention				
Yes	117 (29.4)	34 (8.5)	1.81 (1.14–2.87)	2.4 (1.36–4.21)
No	162 (40.7)	85 (21.4)	1	1
TV is my information source				
Yes	169 (42.5)	70 (17.6)	1.10 (0.69–1.67)	0.78 (0.35–1.35)
No	110 (27.6)	49 (12.3)	1	1
Got information from social media				
Yes	194 (48.7)	65 (16.3)	1.90 (1.22–2.95)	2.51 (1.42–4.53)
No	85 (21.4)	54 (13.6)		1
Presence of IP guideline				
Yes	132 (33.0)	27 (7.0)	3.1 (1.88–4.99)	2.82 (1.64–4.62)
No	147 (37.0)	92 (23.0)		1
High workload				
Yes	112 (28.0)	55 (14.0)	0.78 (0.51–1.20)	0.82 (0.53–1.14)
No	167 (42.0)	64 (16.0)	1	1

### Factors affecting COVID-19 prevention among health workers

HWs from rural areas were 55% times less likely to have good COVID-19 prevention practices than their counterpart HWs (AOR 0.45, 95% CI = 0.31–0.75). The odds of having good COVID-19 prevention was twice among HWs who took training in IP and who had access to IP guideline (AOR = 2.32, 95% CI = 1.35–4.16; AOR = 2.10, 95% CI = 1.21–3.45), respectively. Also, HWs who had good knowledge of COVID-19 were triple times to prevent it compared to HWs who had poor knowledge (AOR = 2.98, 95% CI = 2.15–5.27). The odds of having good preventive practice were twice among HWs who had chronic illnesses than the counterpart HWs. Moreover, HWs who had limited access to PPE, high workload, and HWs from health centers were 58%, 60%, and 60% times less likely to have good COVID-19 prevention practice, respectively (Table 3).

**Table 3:** Factors of COVID-19 prevention among HWs in Amhara Region, Ethiopia, 2020

Variables	HWs practice		COR (95% CI)	AOR (95% CI)
	Good	Poor		
Age				
< 34	110 (27.6)	77 (19.4)	0.77 (0.51–1.16)	0.68 (0.37–1.13)
≥ 34	137 (34.4)	74 (18.6)	1	1
Sex				
Male	138 (34.7)	93 (23.4)	0.79 (0.52–1.93)	1.3 (0.82–2.61)
Female	109 (27.4)	58 (14.6)	1	1
Residence				
Rural	120 (30.2)	105 (26.4)	0.41(0.27–0.63)	0.45 (0.31–0.75)
Urban	127 (32.0)	46 (11.6)	1	1
Working experience				
≤ 5 years	101 (25.4)	58 (14.6)	1.12 (0.73–1.68)	0.89 (0.41–1.40)
>5 years	146 (36.7)	93 (23.4)	1	1
Health facility of HWs				
Health center	128 (32.0)	116 (29.0)	0.32 (0.21–0.51)	0.40 (0.28–0.89)
Hospital	119 (30.0)	35 (9.0)	1	1
Trained in IP				
Yes	115 (29.0)	36 (9.0)	2.78 (1.77–4.37)	2.32 (1.35–4.16)
No	132 (33.0)	115 (29.0)	1	1
Knowledge about COVID-19				
Good	198 (49.7)	81 (20.4)	3.49 (2.23–5.46)	2.98 (2.15–5.27
Poor	49 (12.3)	70 (17.6)	1	1
Use TV as information source				
Yes	146 (36.7)	93 (23.4)	0.85 (0.56–1.29)	1.31 (0.85–2.78)
No	103 (25.9)	56 (14.0)	1	1
Presence of IP guideline				
Yes	117 (29.4)	42 (10.6)	2.34 (1.51–3.61)	2.10 (1.21–3.45)
No	130 (32.6)	109 (27.4)	1	1
Have chronic illnesses				
Yes	46 (11.6)	14 (3.5)	2.24 (1.19–4.23)	2.0 (1.15–3.75)
No	201 (50.5)	137 (34.4)	1	1
Smoke cigarette				
Yes	28 (9.5)	20 (2.5)	0.84 (0.45–1.55)	0.58 (0.24–1.31)
No	219 (52.5)	131 (35.5)	1	1
Drinking alcohol				
Yes	130 (32.7)	49 (12.3)	0.84 (0.53–1.30)	0.64 (0.38–1.26)
No	167 (42.0)	52 (13.0)	1	1
Shortage of PPE				
Yes	93 (23.4)	98 (24.6)	0.33 (0.21–0.50)	0.42 (0.32–0.74)
No	154 (38.7)	53 (13.3)	1	1
Shortage of disinfectants				
Yes	130 (32.7)	156 (39.2)	0.80 (0.52–1.25)	0.60 (0.32–1.20)
No	57 (14.3)	55 (13.8)	1	1
High workload				
Yes	77(19.4)	90 (22.6)	0.31 (0.20–0.67)	0.40 (0.36–0.87)
No	170 (42.7)	61 (15.3)	1	1
Discomfort from wearing PPE				
Yes	125 (31.4)	82 (20.6)	0.86 (0.57–1.30)	0.62 (0.36–1.21)
No	122 (30.7)	69 (17.3)	1	1

## Discussion

This study assessed the knowledge and practice of HWs concerning COVID-19 and identified factors associated with knowledge and infection control practices of HWs about COVID-19. The outputs of this study are crucial to HWs, health facilities, health offices and researchers to halt the spread of COVID-19 and fill literature gap [20, 23, 30]. Because HWs are at the front line in the COVID-19 prevention system, they have the highest risk of acquiring the infection and spreading it to their families and the community [16, 24, 25]. In addition, HWs have faced psychological stress and social stigma because of COVID-19 and their occupation [12, 18, 31].

This study depicted that over two-thirds (70%) HWs had good knowledge about COVID-19. This finding is higher compared to findings from Bale Zone [24] and Addis Ababa [26], Ethiopia where the knowledge of HWs about IP practices in health facilities was 55.4% and 38.6%, respectively. This difference might be related to variations in the study period, study area coverage and the nature of the topic. This study included large area coverage (region level), whereas the former studies were at zonal levels (administrations within a region). When we see the time and nature of the topic, our study is knowledge about COVID-19 which is a timely issue but the former studies were about knowledge on overall IP practice and work safety of health facilities. Thus, COVID-19 has gotten global attention and advertised via social and mass media to inform the population at large.

On the other hand, the current knowledge level was found to be lower compared to former study findings of COVID-19 and IP practices. It was found lower than 81.6% from Gondar University Hospital [25], 86.4% from Dessie Hospital [32], 84% from Bahir Dar City [33], and 84.6% from Debremarkos Town [34]. All the former studies were about knowledge of HWs on the general IP practices but this study is HWs’ knowledge about COVID-19. The time of the study and studied topic might also contribute to this variation. This study is about HWs’ knowledge of COVID-19 that is not well known and fully practiced in rural health facilities due to no diagnostic and treatment services. This might lower the HWs’ knowledge about COVID-19.

Moreover, this finding was found lower than 82.4% knowledge on COVID-19 from Uganda [15], 78.6% from Nigeria [16], 93.2% from Pakistan [30], 89% and 90% from China [35, 36], and 80% from the USA [37]. This variation might be caused by differences in the study area and population, geographic coverage, and number and type of questions used. The current study used large area coverage where most participants were from rural areas that had limited access to information sources, IP practices and COVID-19 diagnosis and support services than the situation in the abroad that included urban health facilities with better access to information sources, IP facilities and COVID-19 prevention practices.

In this study, HWs had 80–85% scores for the causative agent (virus), knowing highest risk population groups, no treatment/vaccine, and prevention mechanisms (isolation, social distance, and handwashing of COVID-19). This is in line with findings from the former studies on COVID-19 [30, 35–37]. HWs however had lower scores (45–76%) for questions related to transmission routes of COVID-19. This is a critical issue that needs special attention from the concerned offices because prevention might be in place if HWs knew well the transmission routes. The low scores might be due to including more HWs from rural health facilities that had limited access to information sources and preventive devices [13, 38, 39].

This study demonstrated the main information sources to HWs where 60% and 65% accessed information about COVID-19 from TV and social media (Facebook, Youtube, Telegram and Twitter), respectively. This is because of easily accessible to most HWs at home and working areas through the mobile internet. This was different from the situation in Saudi Arabia where most of the HWs accessed information about COVID-19 and other infectious diseases form the website of the Ministry of Health [40]. This implied that the Ethiopian Government and the Ministry of Health need to use social media and television to disseminate information to HWs.

This study indicated that the knowledge and practice of HWs were not matching. Only 62% of HWs had good prevention practices towards COVID-19. This implied that more HWs who had good knowledge had poor prevention practices. It might be due to the absence and /or poor quality of PPE and reservation from using PPE due to some discomforts. Thus, priority needs to be given to improve prevention practices parallel to awareness creation and making PPE available. Handwashing and wearing of facemasks and glove were frequently practiced and had up to 82% of scores. Differently, only 29% of HWs always disinfect tables, chairs, other materials and their rooms before and after work. It was incomparable with study findings from Nigeria [16] where the use of disinfectants among HWs was 83.9%. This might be either because of no access to disinfectants or less attention to the values of disinfectants in Ethiopia.

The overall practice score was almost consistent with study findings from Addis Ababa [26] and Wolita Sodo [41], Ethiopia, where the infection prevention practices of HWs were 66.1% and 60.5%, respectively. On the other hand, it was higher than 36.8% from Bale zone [24], 57.4% from Gondar University Hospital [25], 23% from Dessie Town [32], 54.2% from Bahir Dar City [33], 56.8% from Nigeria [16] and 57.3% from Debremarkos Town [34]. This difference might be related to variations in the study period, study topic, presence of IP guideline and PPE materials, access to IP training and commitment of HWs [15, 24, 25].

In contrast, this finding was lower than study findings from Uganda [15], China [35] and Pakistan [30] in which 74%, 89.7% and 88.7% of HWs practiced COVID-19 prevention strategies, respectively. This inconsistency might be related to variations in the geographic area, the incidence of COVID-19, availability of PPE, IP policies among countries, training access, information sources and awareness levels of HWs [24, 42].

Based on the analysis, the rural residence was found to have an inverse association with the knowledge and practice of HWs towards COVID-19. HWs from rural health facilities had less likely odds to have good knowledge and practice (Tables 2 and 3). This might be related to limited access to health information sources such as guidelines, training, and the internet to update themselves. There is also limited access to PPE, washing facilities, isolation rooms and disinfectants [24, 32, 42]. Also, from personal observation, the rural community had less awareness of COVID-19. All these might lead them to have limited knowledge and prevention practices about COVID-19 compared to HWs in urban settings.

Based on the multivariable model, being trained in IP and having IP guidelines were positively associated with the knowledge and prevention practice of HWs towards COVID-19. This was supported by findings from formers studies [15, 25, 26, 42] that reported training was a predictor to improve the knowledge and practice of IP among HWs. The primary aim of training in IP is to improve the knowledge of HWs about preventive mechanisms and how to apply them to prevent infections at working places and accessing the required IP equipment and guidelines. If HWs have IP guidelines and know well how to prevent and the risk of not practicing preventive strategies, they will apply all the possible preventive mechanisms to avoid infections. Most of the time, good knowledge from training and IP guidelines is a predisposing factor for having better infection prevention practices [24, 26, 30].

HWs who used social media as information sources had over double times odd to have good knowledge about COVID-19 compared to the counterpart HWs. This result was supported by former study findings from China [35, 36] and Iran [43] in which the main source of knowledge about infection prevention was using social media. It might be linked to ease of use and access the service using everywhere using mobile internet and social media (Facebook, Youtube, Twitter and others) are have been used worldwide. So, everybody can update his knowledge and information demand using these media. The informants (government and ministry of health) need to assess the media preference of HWs and the community to offer information concerning the COVID-19 and other health-related information effectively.

In this study, health facility type was found to be statistically associated with infection prevention practices of HWs. HWs who worked in HCs were 60% times less likely to practice COVID-19 prevention than hospital health workers. This was supported by previous studies [16, 24, 33, 42] where HWs working in urban and advanced hospitals had better infection prevention practices than the rural and primary care health facilities. This might be attributed to the availability of better training, PPE, IP guidelines, personal commitments and follow-up, and advanced healthcare procedures (surgery) that lead to infection. There might also more COVID-19 suspected cases in hospitals and referral places. All these mandated the hospitals to have relatively better awareness and IP practices among HWs in hospitals.

Moreover, HWs who had good knowledge about COVID-19 were triple times more to have good IP practices. The former studies reported similar findings that good knowledge of HWS about IP was determinant to have good IP practice [15, 26, 30, 33, 41]. It is true that if HWs have better knowledge about IP and its importance, they will possibly apply it in their working areas and make themselves safe from acquiring work-related infections. This indicates that regular update of HWs through training and availing IP guidelines is needed from the health system managers.

Having chronic illnesses among HWs was positively associated with IP practices of HWs. HWs who had chronic illnesses were twice to have good IP practice than HWs who had no history of chronic illnesses. This is linked to the nature of COVID-19 and fears that people with chronic illnesses (DM, hypertension, cardiac problems, renal failure, respiratory problems and others). People with such health problems have been identified as the highest risk groups to acquire COVID-19 and become seriously ill from the infection including loss of life than other people [5–8, 11, 22]. Thus, HWs with such health problems would implement all preventive strategies not to get the infection compared to other HWs.

Furthermore, the shortage of PPE and high workload were negatively associated with the IP practice of HWS (AOR = 0.42, 95% CI = 0.32-0.74, and AOR = 0.40, 95% CI = 0.36–0.87), respectively. The study findings from former studies supported these associations [13, 15, 16, 18, 26]. If there is no access to PPE among HWs, they will not practice IP even if they have adequate knowledge and attitude about IP. In addition to the shortage of PPE, HWs may not apply IP strategies and use PPE properly if they are busy and overloaded with tasks.

### Limitation of the study

Although this study has a wide area coverage, the second largest region in Ethiopia, it has some limitations that might have minimal impact on the study findings and external validity. It was based on online data collection techniques using email and telegram. Some of the health workers might not have access to such services due to limited access to technology, internet service and electric power. Thus, they might not be sampled even if they are important to this study. Also, this study included HWs working only in government health facilities. These might have some limitations in the external validity of the research findings while considering the whole HWs found in the region. Since it is a one-time study, it shared the limitations of a cross-sectional study to establish cause-effect relationships.

## Conclusion

In conclusion, the majority of HWs in the Amhara Region had good knowledge of COVID-19 despite limited prevention practices during the outbreak. Lower age, rural residence, access to training in IP, work with IP guidelines and using social media as information sources were statistically significant factors of HWs’ knowledge about COVID-19. Whereas, rural residence, facility type, presence of IP training and guidelines, knowledge about COVID-19, having chronic illnesses, lack of PPE and high workload were significant factors associated with the IP practice of HWs. Thus, a consistent supply of PPE and improving health workers’ knowledge through training, and making IP guidelines and information sources available are crucial to prevent COVID-19 infection. Also, managing chronic illnesses and balancing the workloads are required to reduce the risk of acquiring COVID-19 infection.

## Supplementary information

**Additional file 1.** Information sheet, Statement of Consent, Questionnaire.

## Data Availability

The datasets analyzed during the current study are available from the corresponding author on reasonable request.

## Abbreviations

AOR: Adjusted odds ratio
CI: Confidence interval
COR: Crude odds ratio
COVID-19: Coronavirus Disease 2019
HCs: Health centers
HWs: Health workers
IP: Infection prevention
PPE: Personal Protective Equipment
