# Supplementary material for: Factors determining the knowledge and prevention practice of healthcare workers towards COVID-19 in Amhara region, Ethiopia: a cross-sectional survey
Source: Trop Med Health. 2020 Aug 20;48:72. doi: 10.1186/s41182-020-00254-3 (PMC7438679; doi:10.1186/s41182-020-00254-3)
Supplement: Supplementary file 1 — Additional file 1. Information sheet, Statement of Consent, Questionnaire. [file 41182_2020_254_MOESM1_ESM.docx]

**Title: Factors determining the knowledge and prevention practice of health workers on COVID-19 infection in Amhara Region, Ethiopia: A cross-sectional survey.**

**Investigator: Mulusew Andualem Asemahagn**

School of Public health, College of Medicine and health sciences, Bahir Dar University, Bahir Dar Ethiopia.

**Information sheet**

Coronavirus Disease 2019 also known as COVID-19 is rapidly expanding to a number of countries globally and is now a pandemic that has claimed over 10 million lives and nearly half million deaths. It has become a public health threat in Ethiopia causing around 5 thousand new cases and 80 deaths as of 23 June 2020. In this case, healthcare workers are at the forefront of the pandemic response and are exposed to dangers that put them at the highest risk of acquiring COVID-19 virus infection. They are facing pathogen exposure, long working hours, psychological distress, fatigue, occupational burnout, and social stigma. Large number of health workers have lost their lives by COVID-19. They have double sources of infections from working places and the community. They are also primary sources to spread the infection to their facilities and the community by taking their infections from various sources. More healthcare workers have lost their lives to COVID-19 virus.

Poor knowledge and practice of health workers can determine their level of prevention towards COVID-19. This will make them to have delayed diagnosis and high spread of the infection to others. Thus, being updated with valid information is critical to health workers to manage their patients safely and keep themselves and their families free from acquiring COVID-19 infection. They also need have better access to proper protective equipment at work places. Thus, the purpose of this study is to assess the knowledge and practices of healthcare workers towards COVID-19 virus infection in the Amhara Region, Ethiopia. This will be important to make decisions on improving infection prevention practices in health facilities through accessing the required materials and trainings.

Data for this study will be collected by online system using your email and telegram addresses. As you know, it is highly secured and no any personal identifier is needed after data collection. No one other than the investigator will have access to your data. You are randomly selected by chance from all list of health workers in the region. You will get nothing incentives because of your participation except being part of the COVID-19 prevention and evidence generation. Participation is fully voluntary including withdrawing from the participation. It will take only 10 - 15 minutes to complete. If you are volunteer to be part of this study, please read, sign and send the following consent form. If you need further clarity, please contact the investigator; Mulusew Andualem Asemahagn at Email: [muler.hi@gmail.com](mailto:muler.hi@gmail.com)

By clicking the next button, I read and gave my consent to take part in this study.

*Required

**Statement of Consent**

I have been asked to participate in the research study. The study has been explained well to me. I understand what the study means to me including what I (the participant) have to go through while in the study. I have had an opportunity to ask questions about the study and have been answered in the best way for me to understand. If there are any other questions that I have to ask later, I will freely approach the study representatives whose contact I have been provided with. I also understand that my participation is voluntary, my data will be confidential with no use of any personal identifies. I have told as my consent can be withdrawn any time in the meantime without any precondition. Thus, I have accepted the offer to be part of this study using my signature.

Participant ID. _________________ Signature_______________ date______________

**Questionnaire**

**Instructions**

1. For each section, respondents are well come to choose all the possible choices/ more than one answer.
2. A required field on each page must be completed to pass to the next question or page. [*****Required]
3. Adequate glove? Mask, sanitizer, etc means: If you have no shortage to your daily use of protective equipment and you have not missed to use since the occurrence of COVID-19 and no a shortage message from your facility.
4. The questionnaire has three sections: A) Socio-demographic (personal related questions) that consisted of 8 questions. B) Risk assessment and information source related questions consisted of 14 questions. C) Knowledge questions about COVID-19 (16 questions) about its causative agent, route of transmission, signs and symptoms, most risky groups and preventive measures. 4). About 11 questions about the preventive practice of health workers from getting COVID-19 infection.

**Note**: To each section, you can select more than one or all the possible choices to the questions.

| **A** | **Socio-demographic related questions** | |
| --- | --- | --- |
| **Sr.N.** | **Questions** | **Response** |
| 1 | Age in years | _______________ |
| 2 | Your sex | □ Male □ Female |
| 3 | Your profession | □ Physician □ Nurse □ Health officer □ Midwifery □ Laboratory |
| 4 | Your residence | □ Rural □ Urban |
| 5 | What is your marital status | □ Single □ Married □ Divorced |
| 6 | How many family members do you have | ____________ |
| 7 | How long have you been working there in years | _____________ |
| 8 | Did you get raining on infection prevention practices in this year | □Yes □ NO |
| **B** | **Risk and information source assessment questions** | |
| 1 | Do you have travel history within two months | □Yes □No |
| 2 | Do you have chronic illnesses (DM, kidney disease, heart diseases, hypertension? … | □Yes □No |
| 3 | Do you have close contact with confirmed cases | □ Yes □ No |
| 4 | Do you smoke cigarette in any amount | □Yes □ No |
| 5 | Do you take alcohol in any amount | □Yes □ No |
| 6 | Do you have internet access (wifiy or mobile data) | □Yes □ No |
| 7 | Do you have TV and/ or radio | □Yes □ No |
| 8 | Do you use consultation of seniors for COIVID-19 virus diagnosis and further update | □Always □ Occasionally □ Never |
| 9 | Do you use social media for information access about COVID-19 | □ Always □ Occasionally □Never |
| 10 | Do you have adequate facemask in your facility | □Yes □ No |
| 11 | Do you have adequate hand rub sanitizer in your facility | □Yes □ No |
| 12 | Do you have adequate disinfectant in your facility | □Yes □ No |
| 13 | Do you have infection prevention guideline/manual in your working area | □Yes □ No |
| 14 | Which of the following lower your prevention practice of COVID-19 virus infection? | □ Shortage of protective equipment  □ High work load for long periods  □ Negligence from my friends/staff  □ Poor quality of protective equipment  □ Discomfort while wearing protective equipment |
| **C** | **Knowledge related questions** | |
| 1 | What is the causative organism of COVID −19? | □ Bacteria □ Virus □ I don’t know |
| 2 | What is/are source of infection of COVID −19? | □ An infected person □ Animals/Birds □ Both of them □ I don’t know |
| 3 | Respiratory droplets during coughing, sneezing from an infected person and close contact with infected person are main transmission routes of COVID-19. | □ True □ False □ I do not know |
| 4 | Contaminated objects and surfaces can transmit COVID-19 | □ True □ False □ I do not know |
| 5 | To which population group COVID-19 virus infection is found to be more severe? | □ Neonates and children  □ Young and middle-aged adults  □ Elderlies and patients with underlying chronic diseases □ I don’t know |
| 6 | What is incubation period of COVID- 19 | □ Less than 7 days □ About 14 days □ About 21 days □ I don’t know |
| 7 | Which of the following are typical symptoms due to COVID- 19? | □ Respiratory symptoms □ Dry cough □ Fever □ Shortness of breath  □ Headaches □ I don’t know |
| 8 | Do you think asymptomatic carriers in subclinical stage can spread the disease? | □ Yes □ No □ I don’t know |
| 9 | There is currently no effective treatment or vaccine for COVID-19, but early symptomatic and supportive treatment can help most patients recover from the infection | □ True □ False □ I do not know |
| 10 | Frequent handwashing with water, soap and alcohol-based hand rub sanitizer prevent COVID-19 infection. | □ True □ False |
| 11 | Keeping social distance as per the standard prevent VOVID-19 virus infection. | □ True □ False |
| 12 | Timely isolation of potentially risky/or confirmed people is important to prevention COVID-19 virus infection | □ True □ False |
| 13 | Wearing facemask or shields is important to prevent acquiring COVID-19 virus infection. | □True □ False |
| 14 | Do you know how to use and dispose personal protective equipment? | □ Yes □ No |
| 15 | Polymerase chain reaction (PCR) is a diagnostic tool to COVID-19 virus infection | □ True □ False □ I don’t know |
| 16 | I know the measures what should do if you develop symptoms and signs suggestive of COVID-19? | □ Yes □ No |
| D | **Prevention practice related questions towards COVID-19** | |
| 1 | Do you cover your mouth and nose with elbow or tissue or handkerchief? | □ Yes □ No |
| 2 | Do you throw the tissue you use safely in a dustbin? | □ Yes □ No |
| 3 | Do you use frequent handwashing with water and soap /or alcohol-based bund rub sterilizer as per recommended? | □ Always □ Occasionally □ Never |
| 4 | Do you routinely wear a facemask or shields at work and outside working places | □ Always □ Occasionally □ Never |
| 5 | Do you wear gloves when you were engaged in patient management | □ Always □ Occasionally □ Never |
| 6 | Do not go to the crowded places | □ Always □ Occasionally □ Never |
| 7 | Do not practice hand shaking or shoulder kissing | □ Always □ Occasionally □ Never |
| 8 | Do you avoid touching your eyes, nose or mouth as far as you can? | □ Always □ Occasionally □ Never |
| 9 | Are you practicing social distancing recommended by the WHO and CDC (2 meters)? | □ Yes □ No |
| 10 | I routinely disinfect of tables, surfaces and working room before and after managing patients | □ True □ False |
| 11 | I have strictly followed a protocol for triage and isolation of suspected COVID-19 cases in my workplace? | □ Trues □ False |
